# Prolonged Syphilitic Taint in the Ova

**Published:** 1900-03-31

**Authors:** 


### PROLONGED SYPHILITIC TAINT IN THE OVA.

The question often arises as to how soon after having had syphilis may a man marry without fear of transmitting the taint to his offspring, and by general consent a probation of two years has come to be regarded as enough to ensure this consummation. In the case of a man this is no doubt true. We must not, however, be so confident in regard to the woman, for long after a woman has ceased to show any symptoms of the disease, and long after she has ceased to be capable of conferring the infection on any one else, she still may produce a syphilitic child—almost as if the ova stored up in the stroma of her ovary had received the infection, and had retained the impulse thus imparted to them long after the general system had reverted to the normal condition, and thrown off all taint. In that wonderful storehouse of interesting experiences, Hutchinson's *Archives of Surgery*, a case is related which appeared lately at the Polyclinic bearing upon this point. A woman brought up two children, one a baby of nine months and the other a boy eight years old, both of them the subject of inherited syphilis. It appeared probable that she had acquired the disease not long before the birth of the elder of the two children, but her symptoms had been of brief duration, and since then she had been in good health. This case would seem to prove that in the case of a mother the ability to transmit syphilis may persist through many years, and Mr. Hutchinson says that he has seen the same kind of proof several times, always, he thinks, in cases in which the mother has herself passed through both primary and secondary stages. In further illustration of the fact he relates the following curious case: "A married man brought me in succession three of his children, all suffering from inherited syphilis. He was always willing, indeed wishful, that they should be treated for that disease, but always denied any knowledge as to how they could have obtained it. He was most positive that he had never had it. His wife never came. Finally he came himself as the patient, with a chancre on one tonsil and an abundant eruption. He had been infected by his own infant, having inadvertently put the nozzle of its bottle into his mouth to start it. He now made a confession. He had known throughout that his wife had been seduced and had suffered from syphilis before he married her. Thus we see that during several years of cohabitation this woman never infected her husband, and we may presume that her secretions were not contagious. She herself appeared to be in excellent health, but she continued to bear syphilitic children. That these children were capable of infecting, and that her husband was in no degree immune, was shown by the latter catching the disease from his child which he had never taken from his wife."

